# cAMP Level Modulates Scleral Collagen Remodeling, a Critical Step in the Development of Myopia

**DOI:** 10.1371/journal.pone.0071441

**Published:** 2013-08-12

**Authors:** Yijin Tao, Miaozhen Pan, Shufeng Liu, Fang Fang, Runxia Lu, Chanyi Lu, Min Zheng, Jianhong An, Hongjia Xu, Fuxin Zhao, Jiang-fan Chen, Jia Qu, Xiangtian Zhou

**Affiliations:** 1 School of Optometry and Ophthalmology and Eye Hospital, Wenzhou Medical College, Wenzhou, Zhejiang, China; 2 State Key Laboratory Cultivation Base and Key Laboratory of Vision Science, Ministry of Health and Zhejiang Provincial Key Laboratory of Ophthalmology and Optometry, Wenzhou, Zhejiang, China; 3 Department of Neurology, Boston University, School of Medicine, Boston, Massachusetts, United States of America; Boston University Goldman School of Dental Medicine, United States of America

## Abstract

The development of myopia is associated with decreased ocular scleral collagen synthesis in humans and animal models. Collagen synthesis is, in part, under the influence of cyclic adenosine monophosphate (cAMP). We investigated the associations between cAMP, myopia development in guinea pigs, and collagen synthesis by human scleral fibroblasts (HSFs). Form-deprived myopia (FDM) was induced by unilateral masking of guinea pig eyes. Scleral cAMP levels increased selectively in the FDM eyes and returned to normal levels after unmasking and recovery. Unilateral subconjunctival treatment with the adenylyl cyclase (AC) activator forskolin resulted in a myopic shift accompanied by reduced collagen mRNA levels, but it did not affect retinal electroretinograms. The AC inhibitor SQ22536 attenuated the progression of FDM. Moreover, forskolin inhibited collagen mRNA levels and collagen secretion by HSFs. The inhibition was reversed by SQ22536. These results demonstrate a critical role of cAMP in control of myopia development. Selective regulation of cAMP to control scleral collagen synthesis may be a novel therapeutic strategy for preventing and treating myopia.

## Introduction

Myopia is the most common visual disorder in the world, with a prevalence estimated to be 25% in the US and Europe and as high as 80% in Asian countries such as China and Japan [1], [2]. It is the fifth most common cause of impaired vision and the seventh most common cause of legal blindness worldwide [3]. The main characteristic morphological change in myopia is elongation of the axial length of the eye, especially evident in the vitreous chamber. This elongation is associated with other pathological changes, including retinal detachment, macular degeneration, cataract, and glaucoma, all of which can cause irreversible vision damage. The current treatment options for myopia are limited.

Although the neurochemical mechanisms underlying myopia are largely unknown, it has been postulated that a visually-evoked signaling cascade in the retina traverses the choroid and triggers scleral remodeling and eye growth [4]. In both human and animal myopia, biomechanical properties of the sclera determine the shape of the eyeball and therefore influence the refractive state of the eye [5]–[7]. Scleral thinning and abnormal scleral fibrils such as fissured and star-shaped fibrils have been observed in human myopic eyes [8]. Myopic development in marmosets, monkeys, and tree shrews is associated with a reduction in scleral thickness and collagen density, in the diameter of scleral collagen fibrils, and in scleral glycosaminoglycan content, particularly at the posterior sclera [9]–[12]. Thus, scleral fibroblasts that synthesize collagen and matrix metalloproteinases play an important role in the maintenance of the extracellular matrix and refractive development.

Cyclic adenosine monophosphate (cAMP) is involved in conduction of visual signals and other neurotransmissions [13], [14] and in control of collagen synthesis [15]–[17]. Previous studies showed that intracellular cAMP increased in response to the adenylyl cyclase (AC) activator forskolin, and inhibited both cell proliferation and collagen synthesis in human pulmonary fibroblasts [15]. Also, increasing cAMP levels by cAMP-elevating agents inhibits the transforming growth factor-β (TGF-β)-stimulated collagen synthesis in cardiac and dermal fibroblasts [16], [17].

Based on the critical involvement of cAMP in the control of collagen synthesis and scleral remodeling during myopia development, we hypothesized that the cAMP levels could underlie myopia development by controlling fibroblast activation and extracellular matrix remodeling. In this study, we first investigated the change of cAMP levels during form-deprivation-induced myopia and recovery in guinea pigs. Next, we used pharmacological manipulation to determine if there is a causal relationship between cAMP levels and the control of refraction and ocular axial dimensions in guinea pigs under normal and form-deprivation environments. Lastly, we studied the effect of cAMP on collagen synthesis by human scleral fibroblasts (HSFs) to explore the molecular basis for cAMP control of myopia.

## Materials and Methods

### Animals and Ethics Statement

Three-week-old pigmented guinea pigs were reared in 12-hour light-dark cycles, with food and water freely available. The treatment and care of animals was conducted according to the Association for Research in Vision and Ophthalmology’s “Statement for the Use of Animals in Ophthalmic and Vision Research”. The protocol for handling animals was in strict accordance with the recommendations and approved by the Wenzhou Medical College Animal Care and Use Committee (Permit Number: WZMCOPT-090316).

### Form Deprivation and Recovery

Form-deprived myopia (FDM) was induced by monocular deprivation using a facemask made of latex. The procedure for preparing and wearing the facemask for two weeks over the right eye has been detailed previously [18]. The recovery group wore the facemask for 2 weeks to induce myopia. The facemask was then removed, and the myopic eye was allowed re-exposure to the normal environment for 2 days.

### Scleral and Retinal cAMP Assay

A ^125^I-cAMP radioimmunoassay (RIA) kit (Institute of Isotopes Ltd, Budapest, Hungary) was used to measure cAMP levels in the sclera and retina. After enucleation, each eye was placed on ice, and the anterior segment (cornea, iris, and crystalline lens) along with the vitreous body were removed from the posterior segment containing the sclera and retina. The sclera and retina were separated from one another, and each was homogenized in 1 ml of acetate buffer (50 mM Na^+^ acetate, 50 mM H^+^ acetate, 4 mM ethylenediaminetetraacetic acid, pH 4.75). Then 2 ml of dehydrated ethanol was added prior to centrifugation at 3,000 rpm for 15 min at 4°C. The supernatants were collected, and the pellets were re-suspended in another 2 ml of 75% ethanol. The re-suspended pellets were again centrifuged as before. The supernatants were collected, pooled with the supernatants from the first spin, and dried at 60°C overnight. The residue was dissolved in 1 ml acetate buffer, and 0.1 ml was assayed according to the manufacturer's protocol. Counts in the re-suspended residue were determined by a gamma scintillation counter (xh6080, Xi'an Nuclear Instrument Factory, Xi’an, Shaanxi, China) for at least 60 seconds and were converted to fmol cAMP/ml.

### Drug Preparation and Injection

Forskolin (Tocris Bioscience, Bristol, UK), an AC activator, was used to elevate the tissue cAMP level. It was dissolved in 3% dimethyl sulfoxide (DMSO) and diluted with 0.9% saline. The vehicle solution was 0.1% DMSO in saline. SQ22536 (Tocris Bioscience, Bristol, UK), an inhibitor of AC, was used to reduce the tissue cAMP level. It was dissolved in double distilled H_2_O and diluted with 0.9% saline. The vehicle solution was 0.1% saline in double distilled H_2_O.

Guinea pigs were randomly assigned to either the normal group or the FDM group. Each was composed of subgroups including non-injection group, vehicle group, and drug-treated group. The right eyes of unanesthetized, restrained normal and FDM animals were injected in the inferior palpebral subconjunctiva with 100 µl of drug or vehicle solution once daily for the period indicated.

### Refractive, Ocular Biometric, and Intraocular Pressure Measurements

Refraction was measured in triplicate by an eccentric infrared photoretinoscope as previously described [19], and a trial lens was used for system calibration. Corneal curvature was measured with a keratometer (OM-4, Topcon, Tokyo, Japan) on which a +8 diopter (D) aspherical lens was attached [18]. Ocular dimensions including the anterior chamber depth, lens thickness, vitreous chamber depth (VCD), and the axial length (AL) were measured with an A-scan ultrasonograph (AVISO Echograph Class I-Type Bat; Quantel Medical, Clermont-Ferrand, France). The A-scan ultrasonography was performed in alert guinea pigs, and the cornea was topically anesthetized with one drop of 0.5% proparacaine hydrochloride (Alcon, Puurs, Belgium). Velocities of sound were assumed as described previously: 1557.5 m/s for aqueous humor, 1723.3 m/s for the lens, and 1540 m/s for the vitreous [20]. Each eye was measured six times. Guinea pig intraocular pressure (IOP) was measured using a TonoVet TV01 Tonometer (TioLat, Helsinki, Finland) at the central cornea of conscious animals according to the manufacturer’s recommendations. The TonoVet takes six measurements deemed reliable by internal software, and then generates and displays the average IOP. In this study, six mean values were obtained from each eye, and the mean of the means treated as a single datum.

### Electroretinograms (ERGs)

After 4 weeks of subconjunctival injections with either vehicle or forskolin, scotopic and photopic ERGs were recorded. ERGs of age-matched, untreated guinea pigs were used as the normal control. Full-field ERGs were recorded with a custom-built Ganzfeld dome connected to a computer-based system (Q450SC UV, Roland Consult, Wiesbaden, Germany). After dark-adaptation for 6 h, scotopic ERGs were recorded between 1 PM and 6 PM, and were followed by the photopic ERGs. White, green (505±6 nm), and blue (470±6 nm) light emitting diodes were used as stimulation light sources for recording the photopic ERGs. All testing was performed in a climate-controlled, electrically isolated dark room under dim red light illumination. The guinea pigs were anesthetized by intraperitoneal administration of ketamine (85 mg/kg) and xylazine (5 mg/kg), and the pupils were dilated with 0.5% tropicamide and 0.5% phenylephrine hydrochloride. A small amount of 2.5% methylcellulose gel was applied to the eye, and a special Ag/AgCl wire loop electrode was placed over the cornea as the active electrode. Needle reference and ground electrodes were placed in the mouth and subcutaneously in the right hind leg, respectively. Recordings were started from the lowest light intensity to the highest. Body temperature was maintained by placing the animals on a 37°C warming pad during the experiment.

### Cell Culture and cAMP Assay

Normal HSFs were established previously as described [21], [22]. All fibroblasts were grown in Dulbecco’s modified Eagle’s medium (DMEM) supplemented with 10% heat-inactivated fetal bovine serum and 2 mM glutamine, and kept in a 37°C incubator with 5% CO_2_. At 80–90% confluence, the cells were plated into 12-well plates to incubate for 24 h. For forskolin and forskolin+SQ22536 treatments, the culture medium was replaced by serum-free DMEM for 24 h before the addition of reagents.

Intracellular cAMP levels were detected by CatchPoint™ Cyclic-AMP Fluorescent Assay Kit (Molecular Devices Corporation, Sunnyvale, CA, USA). HSF cells grown to confluence in serum for 24 h were treated for 30 min with 10 µM forskolin [16], [23]. The cells were lysed using Cell Lysis Buffer (Molecular Devices Corporation) and transferred to 96-well plates coated with a rabbit anti-cAMP antibody. Changes in cAMP levels, measured as pmol of cAMP per mg of protein, were determined in duplicates based on the fluorescence intensity at 590 nm.

### Sircol Collagen Assay

The concentration of soluble collagen in the cell culture medium was measured using the Sircol Collagen Assay Kit (Biocolor Ltd., Carrickfergus, UK). In brief, a 200 µl aliquot of culture medium was added to 1 ml of Sircol dye and mixed in a mechanical shaker at room temperature for 30 min, then centrifuged to pellet the collagen-dye complex. The pellet was dissolved in 0.5 M NaOH to release the collagen-dye complex, and the concentration of collagen was determined by the spectrophotometric absorbance at 540 nm.

### Real-time Reverse Transcription Polymerase Chain Reaction (RT-PCR)

Total RNA was extracted from HSF cultures with Trizol reagent (Invitrogen, Grand Island, NY, USA), and 0.2 µg from each sample was reverse transcribed with M-MLV reverse transcriptase according to the Promega (Promega Corporation, Madison, WI, USA) manufacturer’s instructions. The primers (Table 1) were designed using Primer Express 3.0 software (Applied Biosystems, Foster City, CA, USA). RT-PCR was performed in an Applied Biosystems 7500 Real-Time PCR System using 2×SYBR® Green PCR Master Mix (Applied Biosystems). The results were normalized to the house-keeping gene glyceraldehyde-3-phosphate dehydrogenase.

**Table 1:** Sequences for primers and RT-PCR product length.

Gene	Forward primer (5′-3′)	Reverse primer (5′-3′)	Length
Collagen I	CGAGCGTGGTGTGCAAGGTC	CTGCACCACGTTCACCAGGC	158 bp
Collagen III	ACTCAAGTCTGTTAATGGAC	TATTCTCCACTCTTGAGTTC	120 bp
Collagen V	CAATGGACCCCAAGGACCCA	TTTCTCCCGTGGGACCCTGA	173 bp
GAPDH	GCTCTCTGCTCCTCCTGTTC	GACTCCGACCTTCACCTTCC	100 bp

Following the forskolin injection, the guinea pig eyes were enucleated and prepared as described above. Age-matched, untreated guinea pigs were used as the normal control. The sclera was homogenized in 250 µl of RNA*later®* solution (Ambion, Carlsbad, CA, USA). Total RNA was extracted with RNeasy Fibrous Tissue Mini Kit (Qiagen), and 0.5 µg total RNA from each sample was reverse transcribed with M-MLV reverse transcriptase according to the manufacturer’s instructions (Promega Corporation, Madison, WI, USA). The primers (Table 2) were designed using Primer Express 3.0 software (Applied Biosystems, Foster City, CA, USA). RT-PCR was performed inan Applied Biosystems ViiA™ 7 Real-Time PCR System using 2×SYBR® Green PCR Master Mix (Applied Biosystems). The results were normalized to the house-keeping gene 18S rRNA.

**Table 2:** Sequences for primers and RT-PCR product length.

Gene	Forward primer (5′-3′)	Reverse primer (5′-3′)	Length
Collagen I	CGAGCGTGGTGTGCAAGGTC	CTGCACCACGTTCACCAGGC	158 bp
Collagen III	GTGAAATGGGTCCCGCTGGTAT	GGATCACCTTTGGCACCGTTCT	142 bp
Collagen V	TGAGTTTCTACCCGAAGATGCT	GCAGGAGGGCACATTTCAC	165 bp
18S rRNA	GCAATTATTCCCCATGAACG	GGGACTTAATCAACGCAAGC	68 bp

### Statistical Analysis

Descriptive statistics, including means ± standard error of the means, and statistical tests were determined using the Statistical Package for the Social Sciences (SPSS version 15.0, Chicago, IL, USA). Paired sample t-tests were used to compare biometric data between the experimental and fellow eyes within the same group. Independent sample t-tests were used to compare control and vehicle groups and vehicle and drug groups.

The relative quantity (RQ) of mRNAs and collagen production of HSFs were compared between control and drug-treated groups using independent sample t-tests. Comparisons of effects among groups treated with different concentrations of drugs were performed by one-way analysis of variance (ANOVA) with Bonferroni correction. The RQ of mRNAs for guinea pig scleral collagen was compared between drug-treated and fellow eyes using paired sample t-tests. Independent sample t-tests were used to compare normal and drug-treated eyes.

## Results

### Cyclic-AMP Levels Increased Selectively in Scleras of FDM Eyes

**Figure 1:**
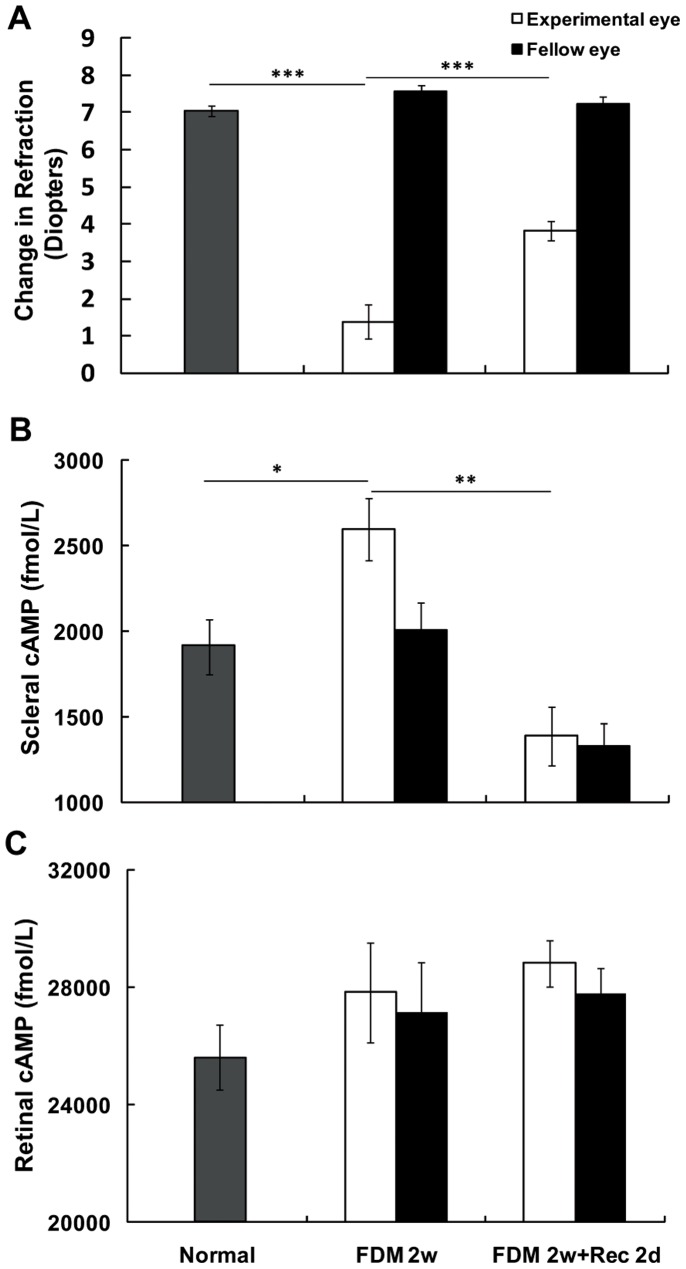
Effect of form-deprived myopia (FDM) on refraction and cAMP levels in the sclera and retina. (A) After 2 weeks of monocular form deprivation, significant myopia was induced in the form-deprived eyes (FDM 2w). Refraction in FDM eyes was significantly increased after 2 days of recovery (FDM 2w+Rec 2d) from FDM (***: p<0.001, independent sample t-test). (B) Scleral cAMP levels in FDM eyes were significantly increased compared to normal eyes (*: p<0.05, independent sample t-test) and fellow eyes (p<0.01, paired-sample t-test). The cAMP level in the deprived eyes was decreased after 2 days of recovery from FDM (**: p<0.01, independent sample t-test). (C) There were no significant changes in retinal cAMP levels between FDM and normal or fellow eyes (Normal, n = 22; FDM 2w, n = 21; FDM 2w+Rec 2d, n = 18).

Two weeks of form deprivation induced significant myopia in guinea pigs (−6.19±0.41D, difference between experimental and fellow eyes, *p*<0.001, paired-sample t-test, Fig. 1A). Refraction in the FDM eyes significantly increased after removal of the facemask for 2 days (p<0.01, independent sample t-test, Fig. 1A). RIA analysis showed that the cAMP levels after 2 weeks of form deprivation were selectively increased in the scleras of FDM eyes in comparison with the fellow eyes (p<0.01, paired-sample t test) and normal eyes (p<0.05, independent sample t test). After 2 days of recovery from FDM, the amount of cAMP in the deprived eyes decreased significantly, reaching the level of fellow eyes (p<0.01, independent sample t test, Fig. 1B). In contrast, the cAMP levels in the retinas of the FDM eyes were not significantly different from the fellow eyes or from normal eyes, and also displayed no significant change after the 2-day recovery from FDM (Fig. 1C).

### Forskolin Induced Myopia in Normal Eyes but had No Effect on Form-deprived Eyes

**Figure 2:**
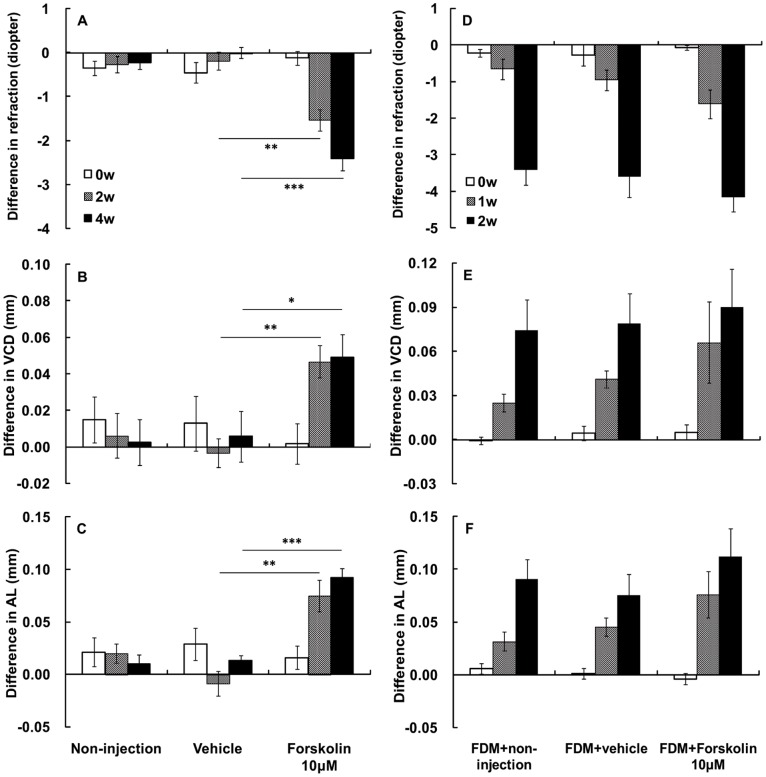
Effect of the adenylyl cyclase activator forskolin on refraction, vitreous chamber depth (VCD), and axial length (AL). (A) Subconjunctival injection of 10 µM forskolin induced the development of myopia in normal guinea pigs compared to non-injected and vehicle-injected controls at 2 and 4 weeks (**: p<0.01, ***: p<0.001, independent sample t-test). (B) Forskolin also induced an increase in vitreous chamber depth (VCD, *: p<0.05, **: p<0.01), and (C) axial length (AL, **: p<0.01, ***: p<0.001) in normal eyes. (D–F) The 1- and 2-week treatments of subconjunctival forskolin had no effects on FDM eyes. (Normal groups: non-injection n = 16, vehicle n = 12, 10 µM forskolin n = 16; FDM groups: FDM+non-injection n = 12, FDM+vehicle n = 9, FDM+10 µM forskolin n = 11).

In normal animals after 2 weeks of daily subconjunctival injection, 10 µM forskolin treatment induced significant myopic refraction (−1.53±0.24D, difference between experimental and fellow eyes) in comparison with the vehicle-treated animals (*p = *0.001, independent sample t-test, Fig. 2A). This change was accompanied by increased VCD (0.05±0.01 mm, *p = *0.001, independent sample t-test, Fig. 2B) and increased AL (0.07±0.01 mm, *p = *0.001, independent sample t-test, Fig. 2C). After 4 weeks of daily injection, forskolin induced greater myopia (−2.42±0.27 D, *p*<0.001, independent sample t-test, Fig. 2A), accompanied by increased VCD (0.05±0.01 mm, *p = *0.029, independent sample t-test, Fig. 2B) and increased AL (0.09±0.01 mm, *p*<0.001, independent sample t-test, Fig. 2C). The myopic shift and associated axial elongation changes were not associated with any significant change in corneal curvature, anterior chamber depth, or lens thickness between forskolin-injected and fellow eyes or between the drug and vehicle-treated groups (data not shown).

We also investigated the effect of forskolin on the progression of FDM. After form deprivation for 2 weeks, forskolin treatment failed to produce any further significant changes in refraction, VCD, or AL in the FDM eyes (Fig. 2D–F). Similarly, forskolin treatment also did not affect corneal curvature, anterior chamber depth, or lens thickness in FDM eyes (data not shown). Taken together, forskolin treatment induced myopic refraction and increased VCD and AL in normal eyes, but had no effect on the myopic shift and associated axial elongation in FDM eyes.

### SQ22536 Reduced Myopia in Form-deprived Eyes but had No Effect on Normal Eyes

**Figure 3:**
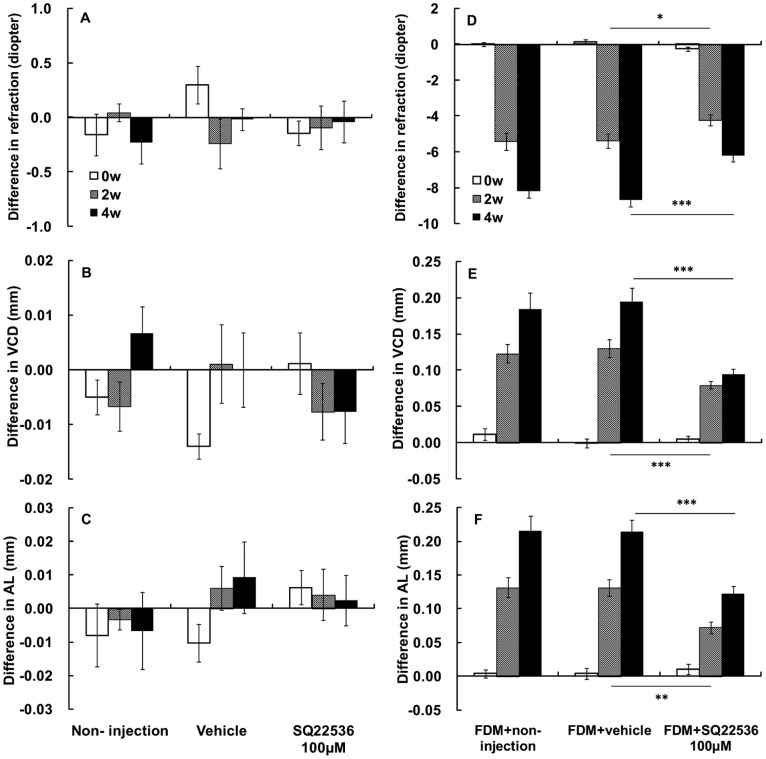
Effect of the adenylyl cyclase inhibitor SQ22536 on refraction, vitreous chamber depth (VCD), and axial length (AL). (A–C) In normal eyes, subconjunctival injection of 100 µM SQ22536 had no significant effect on refraction, vitreous chamber depth (VCD) or axial length (AL). In FDM eyes, SQ22536 reduced (D) refraction (*: p<0.05, ***: p<0.001, independent sample t-test), (E) VCD (***: p<0.001, independent sample t-test), and (F) AL (**: p<0.01, ***: p<0.001, independent sample t-test) in a time-dependent manner. (Normal groups: non-injection n = 11, vehicle n = 14, 100 µM SQ22536 n = 17; FDM groups: FDM+non-injection n = 16, FDM+vehicle n = 17, FDM+100 µM SQ22536 n = 21).

In normal animals, after 2 and 4 weeks of daily subconjunctival injection, 100 µM SQ22536 treatment did not produce any significant change in refraction, VCD, or AL compared to the vehicle-treated groups or to the fellow eyes (Fig. 3A–C). Similarly, SQ22536 treatment also did not affect corneal curvature, anterior chamber depth, or lens thickness compared to the vehicle treatment (data not shown).

We also investigated the effect of SQ22536 on the progression of FDM. After 2 weeks of daily subconjunctival injection, 100 µM SQ22536 treatment significantly reduced the myopic refraction in the FDM eyes compared to the vehicle group (−4.24±0.30 D vs. −5.38±0.39 D, Fig. 3D). SQ22536 treatment also significantly reduced the VCD in the FDM eyes when compared to the vehicle group (0.07±0.01 mm vs. 0.13±0.01 mm, *p = *0.013, independent sample t-test, Fig. 3E). Similarly, SQ22536 treatment significantly reduced the AL in FDM eyes compared to the vehicle group (0.09±0.01 mm vs. 0.13±0.01 mm, *p = *0.004, independent sample t-test, Fig. 2F). After 4 weeks of daily subconjunctival injection, SQ22536 treatment significantly reduced myopic refraction in FDM eyes compared with the vehicle-treated animals (−6.21±0.33D vs. −8.67±0.40 D, *p*<0.001, independent sample t-test, Fig. 3D). The diminished myopia was accompanied with reduced VCD (0.11±0.01 mm vs. 0.19±0.02 mm, *p*<0.001, independent sample t-test, Fig. 3E) and AL (0.12±0.01 mm vs. 0.21±0.02 mm, *p*<0.001, independent sample t-test, Fig. 3F) in FDM eyes compared with the vehicle group. As expected, there were no significant changes in corneal curvature, anterior chamber depth, or lens thickness between SQ22536 injected and fellow eyes or between the SQ22536 and vehicle-treated groups (data not shown). Taken together, SQ22536 had no effect on normal eyes but reduced myopic refraction, VCD, and AL in FDM eyes.

### Forskolin had No Significant Effect on Intraocular Pressure

The IOP was measured in each eye before and after the injection of forskolin at weeks 2 and 4. There were no significant differences in the mean IOPs between the forskolin-injected eyes and the untreated eyes or between the forskolin and vehicle-treated groups at any time point after the injection (data not shown).

### Forskolin had No Significant Influence on Retinal Function

**Figure 4:**
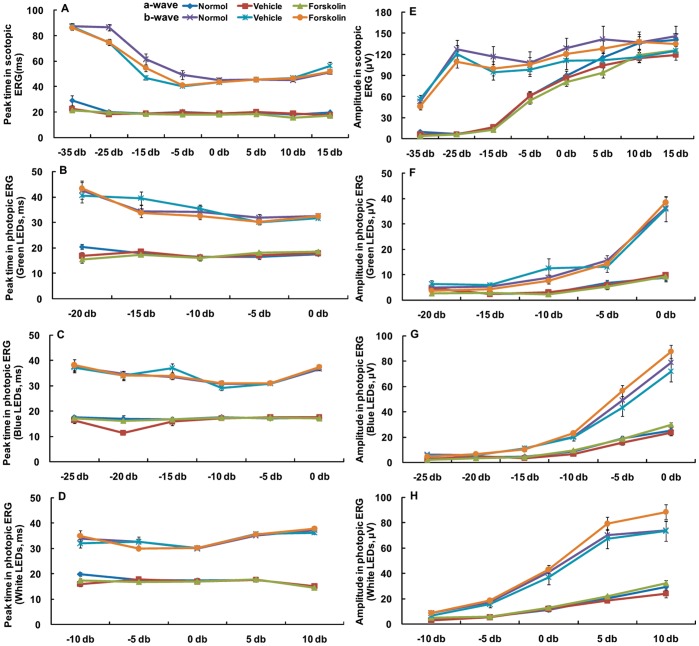
Effect of forskolin on scotopic and photopic ERGs. The scotopic and photopic ERG luminance response curves (peak time, amplitude) for the a-and b-waves were obtained from age-matched normal guinea pigs and forskolin or DMSO injected guinea pigs (normal n = 12, vehicle n = 12, forskolin n = 15). At all stimulus intensities, there were no significant differences between forskolin- and vehicle-treated guinea pigs for either scotopic or photopic ERG parameters. Ordinate, (A–D) peak times in milliseconds (ms) or (E–H) amplitude (µV); Abscissa, flash intensity.

After 4 weeks of injections, retinal function was examined by dark- and light-adapted ERGs. There were no significant differences in either scotopic or photopic ERG parameters at any stimulus intensity between the forskolin and vehicle injection groups or age-matched normal controls (Fig. 4).

### Forskolin Inhibited Collagen Expression in vitro and in vivo

**Figure 5:**
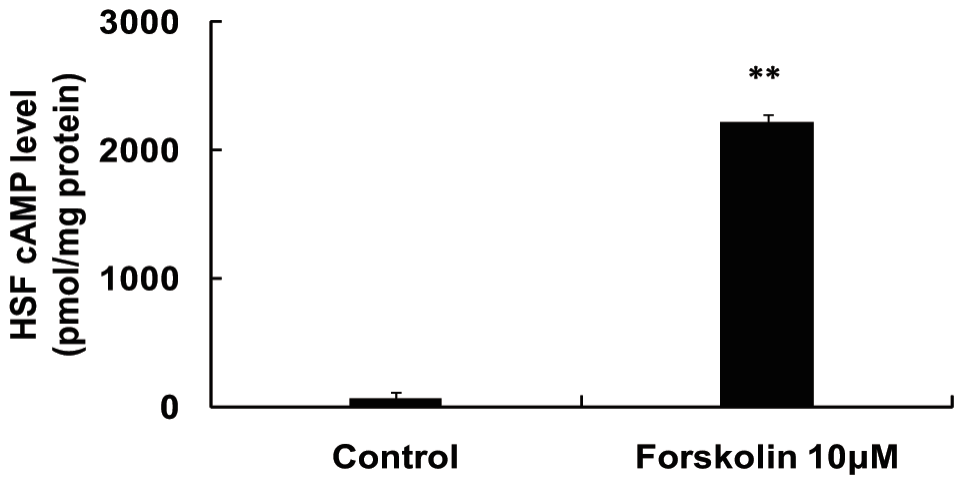
Intracellular cAMP concentration. Cultured human scleral fibroblasts (HSFs) were treated with 10 µM forskolin for 30 min. Forskolin increased the intracellular cAMP levels. (n = 3, **: p<0.01, independent sample t-test).

**Figure 6:**
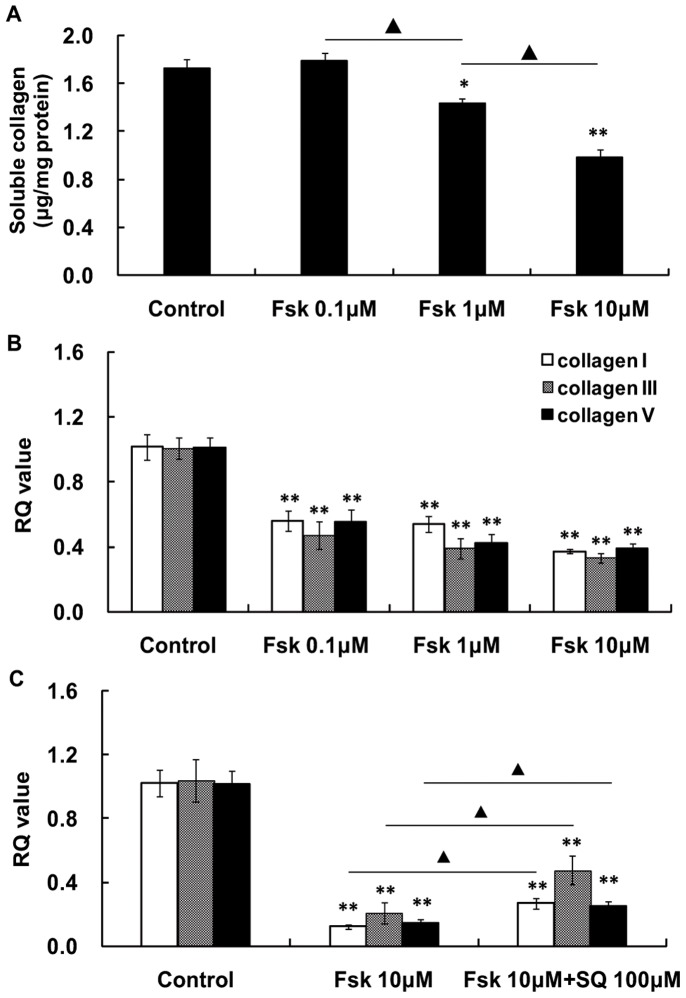
Effect of forskolin and SQ22536 on collagen secretion and mRNA expression in HSFs. (A) Treatment of cultured human scleral fibroblasts (HSFs) for 24 h with forskolin significantly decreased the total soluble collagen recovered in the culture medium in a concentration-dependent manner (*: p<0.05, **: p<0.01, ANOVA, n = 6; ▴: p<0.01, ANOVA, n = 6). (B) Treatment with forskolin for 24 h significantly decreased the expression of mRNAs for collagens I, III, and V. Forskolin reduced the mRNA level in a concentration-dependent manner. (*: p<0.05, **: p<0.01, ANOVA, n = 6). (C) The reductions in collagen I, III, and V mRNA expression levels by forskolin were significantly decreased by SQ22536 treatment. (**: p<0.01, independent sample t-test, n = 6; ▴: p<0.05, independent sample t-test, n = 6).

Following treatment with 10 µM forskolin for 30 min, the intracellular cAMP level of the HSFs increased significantly (Fig. 5). Treatment with 0.1 µM to 10 µM forskolin reduced the total soluble collagen secreted by cultured HSFs in a concentration-dependent manner (Fig. 6A). Forskolin (0.1 µM to 10 µM) also significantly inhibited in a concentration-dependent manner HSF expression of collagen I, III, and V mRNAs (Fig. 6B). Conversely, 100 µM SQ22536 reversed or blunted the forskolin-induced reduction of the collagen I, III, and V mRNA levels that were decreased by forskolin (Fig. 6C).

**Figure 7:**
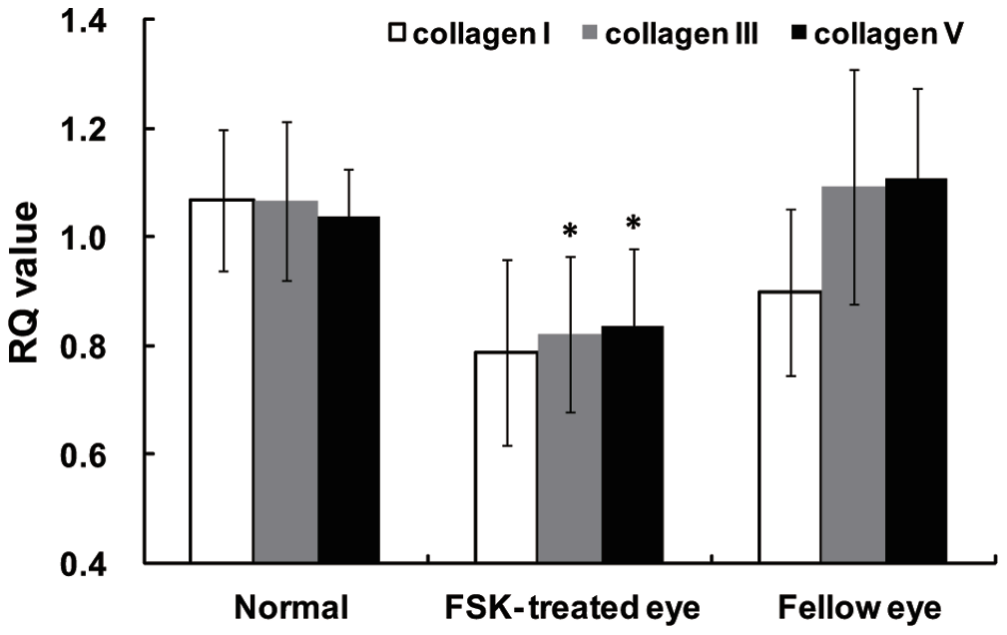
Effect of forskolin on collagen mRNA expression in guinea pig sclera. Injection of forskolin for 1 week significantly decreased the expression of mRNAs for collagens III and V in the experimental eyes. Collagen I mRNA levels also showed a tendency to decrease. (*: p<0.05, paired sample t-test, normal n = 10, forskolin-treated and fellow eyes n = 10).

Furthermore, we also examined the effect of subconjunctival injection of forskolin for one week on collagen I, III, and V mRNA expression in intact animals. We determined by qPCR analysis the expressions of collagen I, III, and V mRNAs in guinea pig sclera after forskolin treatment. We found that collagen III and V mRNA levels were significantly decreased in the forskolin-treated eyes compared with the fellow eyes. Collagen I mRNA levels tended to decrease but the change was not statistically significant after forskolin treatment (Fig. 7).

## Discussion

### The cAMP Level in Sclera Plays a Critical Role in Control of Development of Myopia

The most interesting result of this study is the demonstration of the critical role that cAMP plays in the development of myopia by controlling scleral remodeling and collagen synthesis. Specifically, we showed that pharmacological elevation of the cAMP level by the AC activator forskolin induced axial elongation and refractive myopic shift in normal guinea pig eyes, but had no effect on the FDM eyes. Induction of myopia by the forskolin-induced increase in cAMP level argues that the cAMP level is critical to normal development of visual growth during postnatal development. Similar to form-deprivation (Fig. 2) or hyperopic-defocus [24], an above-normal level of cAMP alone is sufficient to induce myopic changes. This pharmacological finding establishes a causal role of cAMP level in development of myopia. To the best of our knowledge, our results provide the first direct evidence that increases in cAMP level in the eye can cause myopia.

Importantly, ACs and cAMP are widespread in ocular tissues. Non-selective AC drugs could lead to side-effects, such as ocular hypertension or retinal function disorder. We showed that pharmacological reduction of cAMP level with the AC inhibitor SQ22536 selectively inhibited FDM, but had no effect on normal guinea pig eyes. This suggests that FDM eyes with high levels of scleral cAMP are sensitive to AC inhibition but not activation. An important therapeutic implication of our findings is that targeted approaches to reduce cAMP levels in the eye may selectively counteract myopic pathogenesis without affecting normal visual development. Because the onset of myopia occurs mostly in children when normal eye growth and myopic pathogenesis are taking place at the same time, a drug that can distinguish between these two processes will be particularly beneficial. Our data provide strong support for the development of therapeutic strategies for treating myopia that target “super-normal levels” of cAMP. Several AC isoforms are therapeutic targets in brain and heart diseases. For example, AC1 and AC2 are stimulatory targets for treating Alzheimer’s and other neurodegenerative diseases [25], [26], and activation of AC5 and AC6 plays important roles in improving the function of the failing heart [27]–[29]. Thus, investigations into the specific AC isoform(s) and selective inhibitor(s) that specifically decrease cAMP levels in the sclera might lead to development of a novel pharmacological therapy for myopia.

The critical role of the cAMP levels in myopia is consistent with the involvement of scleral remodeling in development of myopia and cAMP as a negative regulator of fibroblast activation and differentiation [4], [17]. In fact, the cAMP pathway may be a common mechanism underlying myopia development, and provides a plausible explanation for the previous finding that several G-protein coupled receptors modify FDM. For example, the activation of D2 dopamine receptor attenuated FDM [30]. Since the D2 receptor is a G_i_-coupled receptor, most likely the effect of D2 activation is achieved through inhibition of AC and reduction of cAMP accumulation [31], rather than due to disturbance of neurotransmitter release [31].

The non-selective and M1-selective muscarinic antagonists, e.g. atropine and pirenzepine respectively, effectively prevent the axial elongation and myopic shift induced by form-deprivation in animal models [32]–[34]. They also halt childhood myopia development [35]–[37]. The expression of the muscarinic M1 subtype is increased in the posterior sclera of FDM eyes in guinea pigs [38]. Levels of cAMP are also regulated by muscarinic receptors, and muscarinic M1 receptors can couple with G_s_ mediating the stimulation of AC activity and cAMP accumulation [39]. Thus, it is possible that atropine and other muscarinic antagonists affect the development of myopia by controlling cAMP levels in the sclera. However, G-protein coupled receptors may modulate myopia by signaling pathways other than cAMP [40]. For example, in contradiction to the predicted results, genetic deletion of the G_s_-coupled adenosine A_2A_ receptor (A_2A_R) and the consequent reduction in cAMP level in fact induces, rather than attenuates, myopia in mice [41].This is accompanied by the finding that A_2A_R activation by the agonist CGS21680 induces expression of mRNAs for collagens I, III, and V and increases production of soluble collagen in cultured HSFs [41]. A_2A_R activation signals through multiple pathways, such as src, ERK1/2, and p38 MAPK, to modulate collagen synthesis [42]. Therefore we speculate that A_2A_Rs may signal through complex pathways to affect collagen synthesis and scleral remodeling and thereby affect myopia development.

### Scleral Remodeling Represents the Main Locus whereby the cAMP Levels Affect Myopia Development by Modulation of Collagen Synthesis

The exact mechanism by which the cAMP levelcontrols myopia development is not entirely clear. Cyclic AMP is present in various ocular tissues, such as the cornea, iris-ciliary body, and sclera [43]. Increased anterior chamber cAMP is associated with increased outflow facility for aqueous humor that, in turn, decreases the IOP [44], [45]. Higher IOP is postulated as a potential mechanical factor for the development of myopia. However, mixed results have been reported, with some studies showing that higher IOP is associated with myopia [46], [47], while others found no significant associations between them [48]–[50]. For example, studies in tree shrew and chick eyes reported that the weakened biomechanical properties of the sclera in myopia reduce the resistance of eye to elongation under the normal IOP [6], [51]. The present study detected no difference in IOP among the normal, vehicle- and forskolin-treated guinea pigs. Consistent with this, there was no significant change in anterior chamber depth and corneal curvature after forskolin or SQ22536 treatment in either normal or FDM guinea pigs. Thus, manipulation of cAMP level by subconjunctival injection did not affect the IOP or the structure of anterior segment.

Our study provides the mechanistic evidence that localizes the sclera as the locus of myopic changes. In guinea pig eyes, the cAMP pathway modified development of myopia by controlling scleral remodeling and collagen synthesis. This notion is supported by three lines of evidences: First, cAMP levels were selectively increased in the scleras, but not retinas, after form deprivation, and they returned to the normal level after 2 days of FDM recovery. This suggests that the sclera represents the main locus where the cAMP level controls myopia development.

The second line of evidence that the sclera is the locus of myopic changes is that subconjunctival injection of forskolin did not affect the ERG parameters of guinea pig eyes. Electroretinography is an effective test of retinal function. Forskolin-elevated cAMP and cAMP analogues delivered by intravitreal perfusion increase a-, b- and c-wave amplitudes in chick and rabbit eyes 52–54. However, the absence of retinal response to subconjunctival forskolin indicated that the development of myopia in guinea pig eyes was independent of any changes in retinal cAMP levels or functions. It is likely that compared to the intravitreal perfusion of forskolin by Jarkman [52] significantly less forskolin permeated through sclera to the retina in this study. Our previous study found that only approximately 1/10,000 of the amount of agent (apomorphine) subconjunctivally injected actually reaches the vitreous [55]. We speculate that forskolin after subconjunctival injection probably acted mainly on the sclera, and thus had no significant influence on retinal function. This finding, together with the selective increase in cAMP in sclera of FDM eyes, suggests that the sclera, with fibroblasts as the main cellular components, is probably the principal locus whereby the cAMP plays a critical role in control of development of myopia.

The third line of evidence that the sclera is the locus of myopic changes is that forskolin treatment reduced the expression of mRNA for collagens III and V in guinea pig sclera, and it tended to reduce the expression for collagen I mRNA. Consistently, forskolin-elevated cAMP levels reduced the amount of the total soluble collagen produced and the expression of mRNA for collagens I, III and V in cultured HSFs. The AC inhibitor SQ22536 blunts the elevation of cAMP content caused by forskolin [56]. Consequently in the current study, the decreased collagen expression by forskolin treatment was partly reversed by SQ22536 treatment. The reduction of collagen I and V by forskolin was consistent with the findings in other human fibroblasts, including dermal, cardiac, and pulmonary fibroblasts [15]–[17]. Of note, the regulation of collagen III expression by forskolin appears to be cell-type specific with a reported increase in pulmonary fibroblasts [15], but a reduction in cultured fibroblasts (present study) as well as in cardiac fibroblasts [16] and Schwann cells [57]. Myopia is characterized by scleral thinning and decreased scleral collagen synthesis [4], [7], [12]. In previous studies, the scleral thinning in myopic eyes of human donors was detected many years after the myopia developed, and in animal studies, the myopia was present for several months. Thus in those studies, it is not clear if the changes in the scleral collagen were consequences or causes of the myopia. In the present study, we showed that the alteration of cAMP levels regulated the development of refraction and the progression of myopia via control over sclera collagen synthesis.

Taken together, the selective increase of scleral cAMP, lack of forskolin-induced change in ERG (i.e., retinal function), and robust modulation of collagen synthesis in HSFs by forskolin lead us to propose that scleral remodeling represents the main locus whereby cAMP regulates myopia development via control of the sclera collagen synthesis.

In summary, using ophthalmological and pharmacological manipulation coupled with optometric measurements, these studies uncovered a critical role of the cAMP level in control of myopia development. Specifically, an abnormally high level of cAMP induced by forskolin is sufficient to induce myopia similar to FDM. Increased cAMP levels result in the myopic shift in guinea pigs, probably by inhibiting scleral collagen synthesis. Moreover, the AC inhibitor SQ22536 can selectively attenuate the progression of FDM without affecting normal visual growth. Thus we hypothesize that inhibition of scleral collagen synthesis is the cause of myopia development. These findings suggest that targeting the cAMP control of collagen synthesis and scleral remodeling may be a novel therapeutic strategy for preventing and treating myopia.
